# Enhanced microglial dynamics in the amyloid plaque microenvironment contributes to cognitive resilience in Alzheimer’s disease

**DOI:** 10.1002/alz.091631

**Published:** 2025-01-03

**Authors:** Nur Jury, Javier Redding, Yanwen You, Pablo Martinez, Hande Karahan, Enrique Chimal Juarez, Travis S Johnson, Jie Zhang, Jungsu Kim, Juan C Troncoso, Cristian A Lasagna Reeves

**Affiliations:** ^1^ Stark Neurosciences Research Institute, Indiana University School of Medicine, Indianapolis, IN USA; ^2^ Johns Hopkins University School of Medicine, Baltimore, MD USA; ^3^ Department of Biostatistics and Health Data Science, Indiana University School of Medicine, Indianapolis, IN USA; ^4^ Center for Comp. Biology and Bioinformatics, Indiana University, School of Medicine, Indianapolis, IN USA; ^5^ Center for Computational Biology and Bioinformatics, Indiana University School of Medicine, Indianapolis, IN USA

## Abstract

**Background:**

Asymptomatic Alzheimer’s disease (AsymAD) refers to individuals with preserved cognition but identifiable Alzheimer’s disease (AD) brain pathology, including beta‐amyloid (Aβ) deposits, neuritic plaques and neurofibrillary tangles upon autopsy. Unlike AD cases, AsymAD exhibits low neuroinflammation and fewer soluble pathological tau species at synaptic levels. However, the link between these observations and the ability to counteract AD pathology is not fully understood. Evidence from AD mice models suggests that the plaque microenvironment significantly influences Aβ plaque‐associated tau pathogenesis. In this study, we investigated the postmortem brains of a cohort of AsymAD cases to gain insight into the mechanisms underlying resilience to AD pathology and cognitive decline.

**Method:**

We conducted a detailed histological and biochemical analysis using postmortem brain samples from age‐matched controls (N = 13), AD (N = 19), and AsymAD subjects (N = 17). In fixed brain tissue, we performed the GeoMx whole spatial transcriptome atlas to compare the gene expression within the Aβ‐plaque microenvironment in AsymAD versus AD cases. To further explore the mechanisms insights of our findings we used human microglial cells.

**Result:**

Our findings showed that AsymAD cases exhibit an enrichment of core plaques and decreased filamentous plaque accumulation with increased surrounding microglia. Less pathological tau aggregation in dystrophic neurites was found in AsymAD versus AD brains, and tau seeding activity was comparable to that in healthy brains. To further characterize the plaque niche, we used spatial transcriptomics, finding an increase in components of the actin‐based motility pathways within the microglia surrounding amyloid plaques in AsymAD brains. Ongoing mechanistic experiments *in vitro* aim to elucidate the role of this pathway in microglial response to Aβ.

**Conclusion:**

Our findings indicate that the amyloid‐plaque microenvironment in AsymAD brains is characterized by microglia with highly efficient actin‐based cell motility mechanisms and decreased tau seeding versus that observed in AD brains. These two mechanisms can potentially protect against the toxic cascade initiated by Aβ, preserving brain health, and slowing AD pathology progression.

